# An Autobiographical Report of Gout in a Patient Unable to Tolerate Conventional Therapy: The Unbearable Pain of Podagra

**DOI:** 10.7759/cureus.78343

**Published:** 2025-02-01

**Authors:** Philip R Cohen

**Affiliations:** 1 Dermatology, University of California Davis Medical Center, Sacramento, USA; 2 Dermatology, Touro University California College of Osteopathic Medicine, Vallejo, USA; 3 Maples Center for Forensic Medicine, University of Florida College of Medicine, Gainesville, USA

**Keywords:** allopurinol, colchicine, crystal, febuxostat, gout, great, medicine, podagra, toe, vitamin

## Abstract

Gout is typically characterized by hyperuricemia and monosodium urate deposition in and around the joints. In individuals affected by gout, the condition can progress through the following phases: asymptomatic hyperuricemia, monosodium urate crystal deposition, acute gout, intercritical period, and chronic gout. Two of the following are required to establish the diagnosis of gout: at least two attacks, observation or a history of podagra or the presence of a tophus, and prompt response after starting treatment with colchicine. A 65-year-old man whose acute gout attack presented with a warm, exquisitely tender, erythematous, area of swelling that affected the first metatarsal of his left great toe (podagra) is described. A short oral course of prednisone treatment successfully managed his acute gout attack. His subsequent workup revealed a slightly elevated serum uric acid level and bilateral gout-related bone changes that were demonstrated on the roentgenograms of his feet. Several systemic medications are available for the management of acute and chronic gout and to prevent the recurrence of gout. However, for individuals who cannot tolerate or are unwilling to use these drugs, an approach that incorporates alternative and complementary medicine may be considered. The patient was not able to be treated with the standard systemic medications that are usually used for individuals with gout. He decided to take vitamin C (at a daily dose alternating from 500 mg to 1000 mg) after his acute attack resolved, since vitamin C has been demonstrated to lower serum uric acid levels and reduce the risk of gout. After a follow-up period of nearly one year, he has not had a recurrent acute gout attack. In conclusion, several systemic medications are recommended for the treatment and prevention of gout; however, for those individuals in whom the standard drugs for managing gout are either ineffective or contraindicated, alternative and complementary medicines may be used for the management of gout.

## Introduction

Gout is a metabolic disorder of uric acid metabolism. Elevated levels of uric acid are frequently present in the blood. Deposits of urate crystals (tophi) can occur in the skin [1].

Several famous people have been affected by gout. Some of them include Benjamin Franklin (American inventor and statesman), Leonardo da Vinci (Italian architect, engineer, painter, and sculptor), Luciano Pavarotti (Italian opera singer), Ludwig Van Beethoven (German composer and pianist), and Sir Isaac Newton (English mathematician and physicist) [2]. Indeed, the renowned *Tyrannosaurus* rex “Sue” from Hell Creek Formation, South Dakota, whose skeleton is currently located in the Field Museum of Natural History in Chicago, IL, USA, is postulated to have suffered from gout [3].

A 65-year-old man who developed an acute attack of gout that had a classical clinical presentation of podagra is described. The acute attack resolved after treatment with oral prednisone. He could not tolerate allopurinol and colchicine; he has been taking vitamin C daily. The management of gout is summarized, and considerations of alternative treatments for individuals who cannot tolerate mainstream drug therapy are discussed.

## Case presentation

A 65-year-old man presented in February 2024 with acute onset of an exquisitely tender and swollen left great toe. He had a past medical history of atrial flutter (which was treated with atrial ablation), an ischemic stroke (which presented with isolated dysarthria and was attributed to the new atrial arrhythmia), hyperlipidemia, hypertension, and lumbar spinal stenosis (requiring back surgery). His medications include apixaban 5 mg twice daily, ezetimibe 10 mg daily, metoprolol succinate ER 25 mg daily, and valsartan 80 mg each evening. He is a nonsmoker and allergic to sulfa drugs (trimethoprim/sulfamethoxazole); none of his relatives have gout.

The man may have had one or two similar prior episodes of severe left toe pain and swelling during the past two years, which eventually resolved spontaneously; he had no history of nephrolithiasis. His diet consisted of red meat at least every other week; he never ate organ meat, such as livers. He rarely consumed shrimp or shellfish. He did not drink full-sugar soda; however, he would regularly drink between 12 and 24 cans (each being 12 ounces) of diet soda each week. He did not change his diet since he experienced the toe pain.

He used to enjoy two or three cans of beer (12 ounces per can) and/or one or two glasses of wine (three ounces per glass) on Fridays, Saturdays, and Sundays. After his stroke on July 27, 2023, he discontinued all alcohol.

Before and after developing toe pain, he continued to maintain an active lifestyle. He would walk five miles three times each week in approximately 1 hour and 40 minutes. On other days, he would swim a mile using freestyle in about 47-54 minutes.

He noticed the area proximal to his left great toe was becoming tender during the day on Friday, February 23, 2024. When he took his sneakers off, he noted that the location was warm to touch, sore, and swollen. He did not recall traumatizing the site and had not experienced any insect bites to his left foot. He was having great difficulty walking and applying any pressure when he put his left foot on the floor.

When he went to bed, he took two 625-mg tablets of acetaminophen; a non-steroidal anti-inflammatory drug (NSAID) was contraindicated since he was on a blood thinner. Acetaminophen did not provide any pain relief. He was hardly able to sleep; he could not even tolerate the weight of the bed sheet on his left great toe.

**Figure 1:**
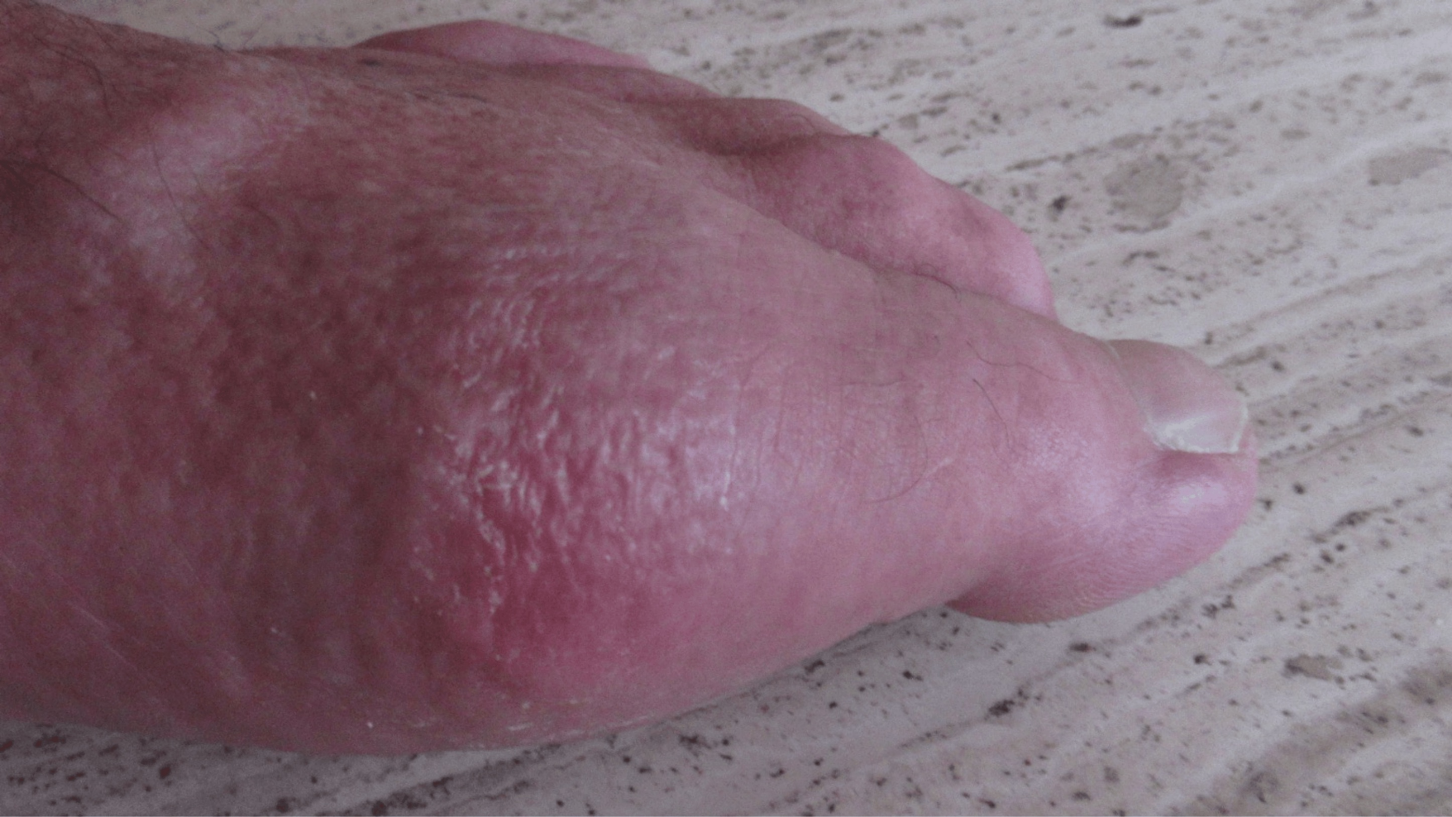
Acute gout presenting as podagra of the left foot. Side view of the distal left foot showing extremely tender, warm, erythematous, massive swelling including the left great toe.

**Figure 2:**
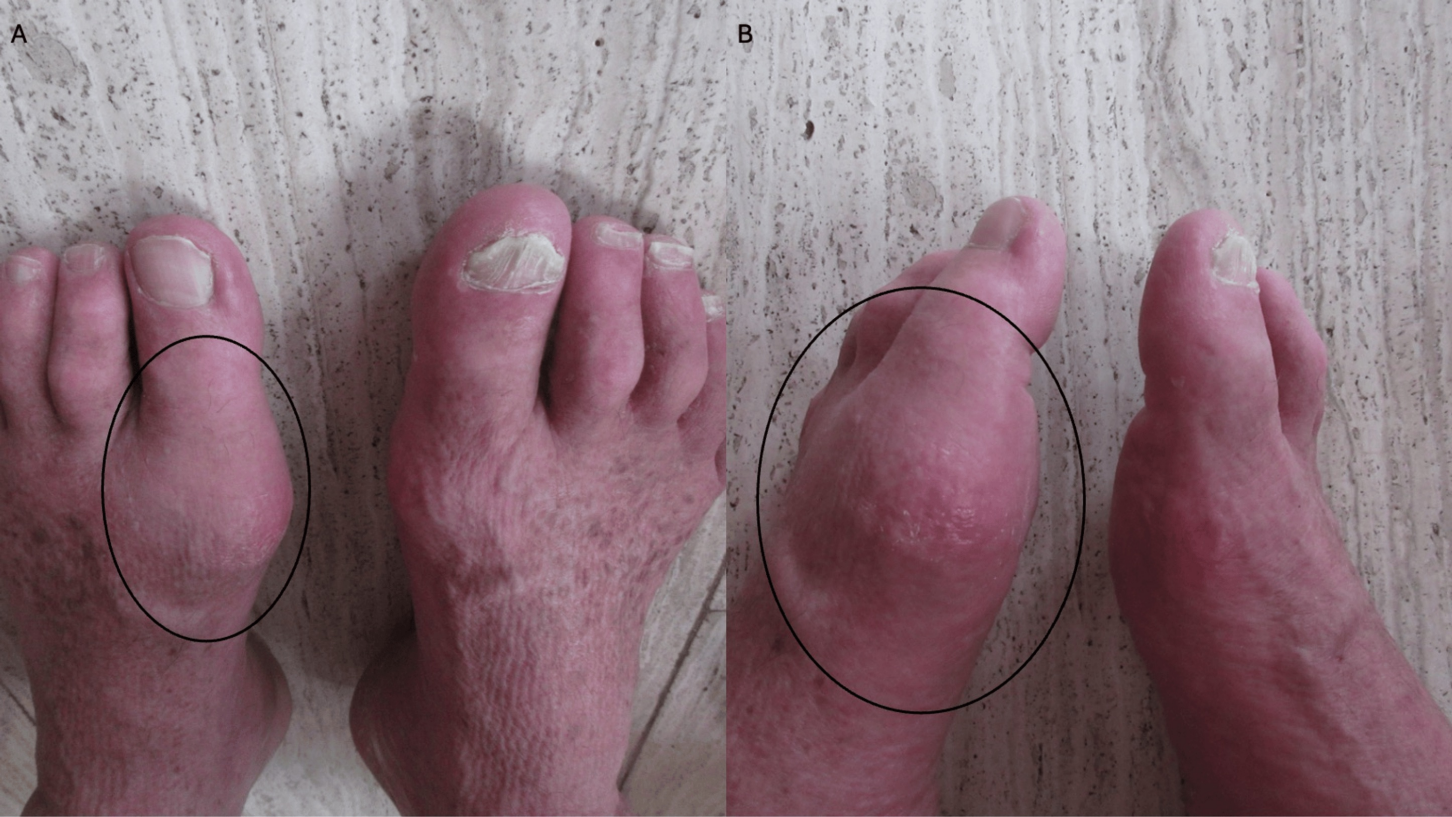
Comparison of the acute gout attack involving the left foot and the non-affected right foot. Top view (A) and side view (B) of the feet. The black hollow oval shows the area of inflammation and edema on the distal left foot.

The following morning, the medial side of his distal foot was extremely swollen (Figure 1 and Figure 2). The area overlying and adjacent to the left metatarsal bone of the great toe was markedly enlarged. It was bright red and hot. It was extraordinarily painful; indeed, the pain was unbearable. He could not tolerate having his foot touched; he could not put on a sock. He could only wear open sandals which did not contact the area.

**Figure 3:**
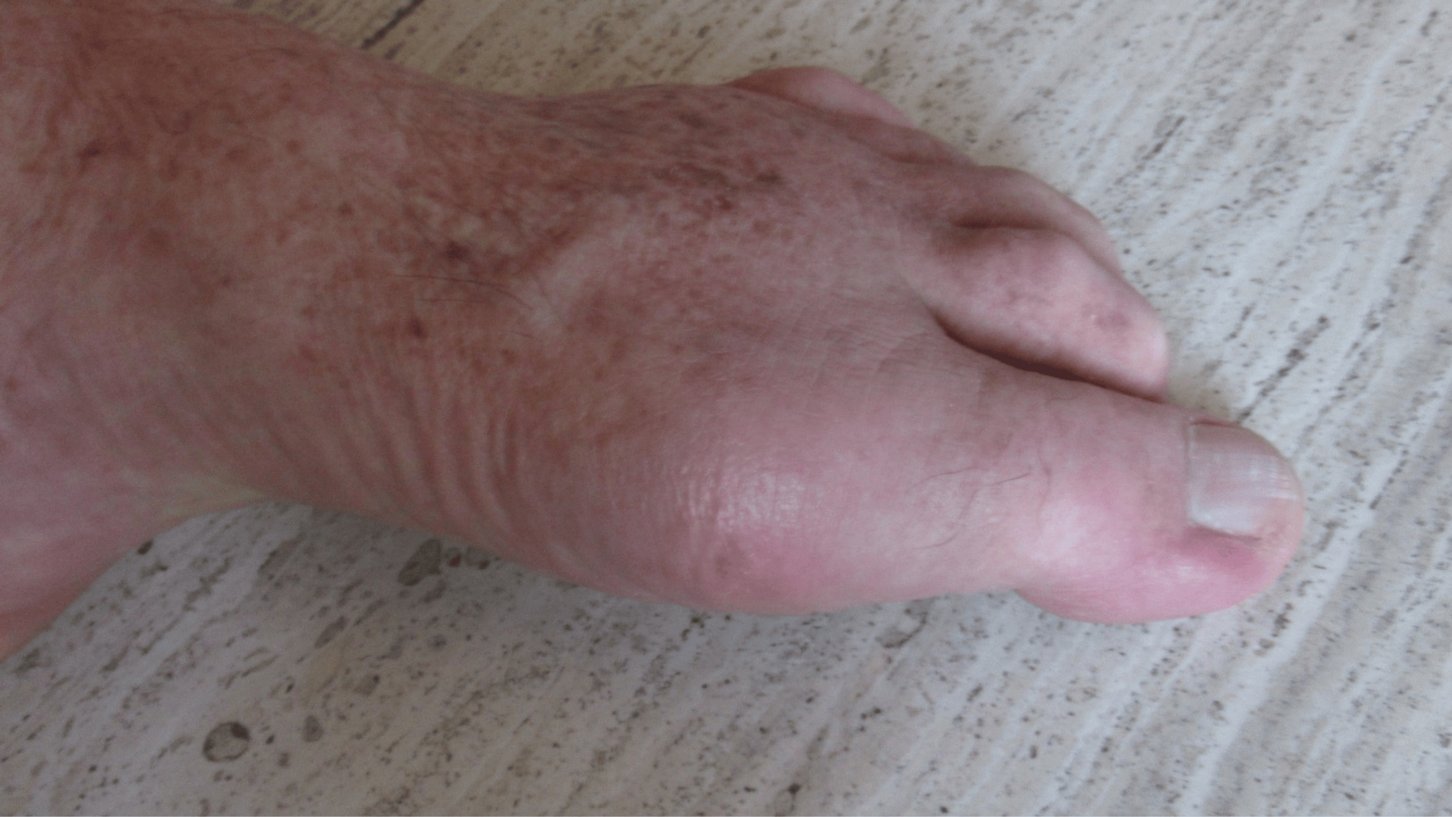
Partial resolution of the acute gout attack affecting the left foot. Side view of the distal left foot showing that the redness and swelling had significantly improved; the pain and warmth were gone.

**Figure 4:**
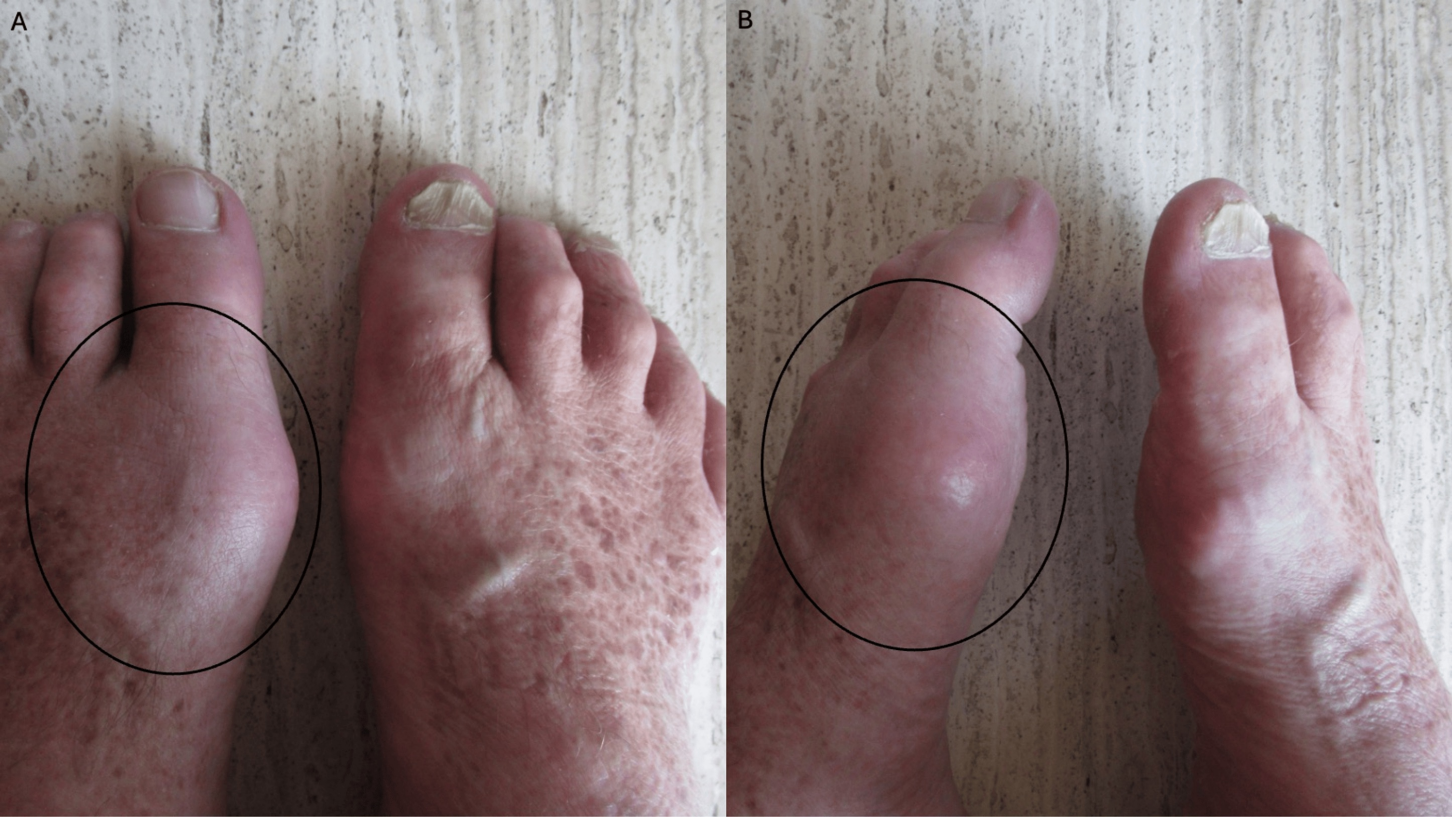
Comparison of resolution of acute gout attack on the left foot and the non-affected right foot. Top view (A) and side view (B) of the feet. The black hollow oval shows the previously affected area of erythema and swelling on the distal left foot.

He had some prednisone in his home. On Saturday, he took 50 mg of prednisone; he decreased the dose by 10 mg each of the subsequent days. By Saturday evening, the left great toe pain had decreased. The swelling and tenderness of his left foot continued to progressively diminish over the next three to four days (Figure 3 and Figure 4). By Friday, March 1, 2024, the swelling and erythema had completely resolved.

He scheduled an appointment with a rheumatologist; the next available appointment was more than six weeks later. On April 18, 2024, laboratory studies were performed; his uric acid was elevated at 7.9 mg/dL (normal range, 3.4-7.0 mg/dL). He was also evaluated for HLA-B5801, which was not present. All remaining laboratory studies (such as complete blood cell counts and platelets, serum chemistries, and liver function tests) were normal.

**Figure 5:**
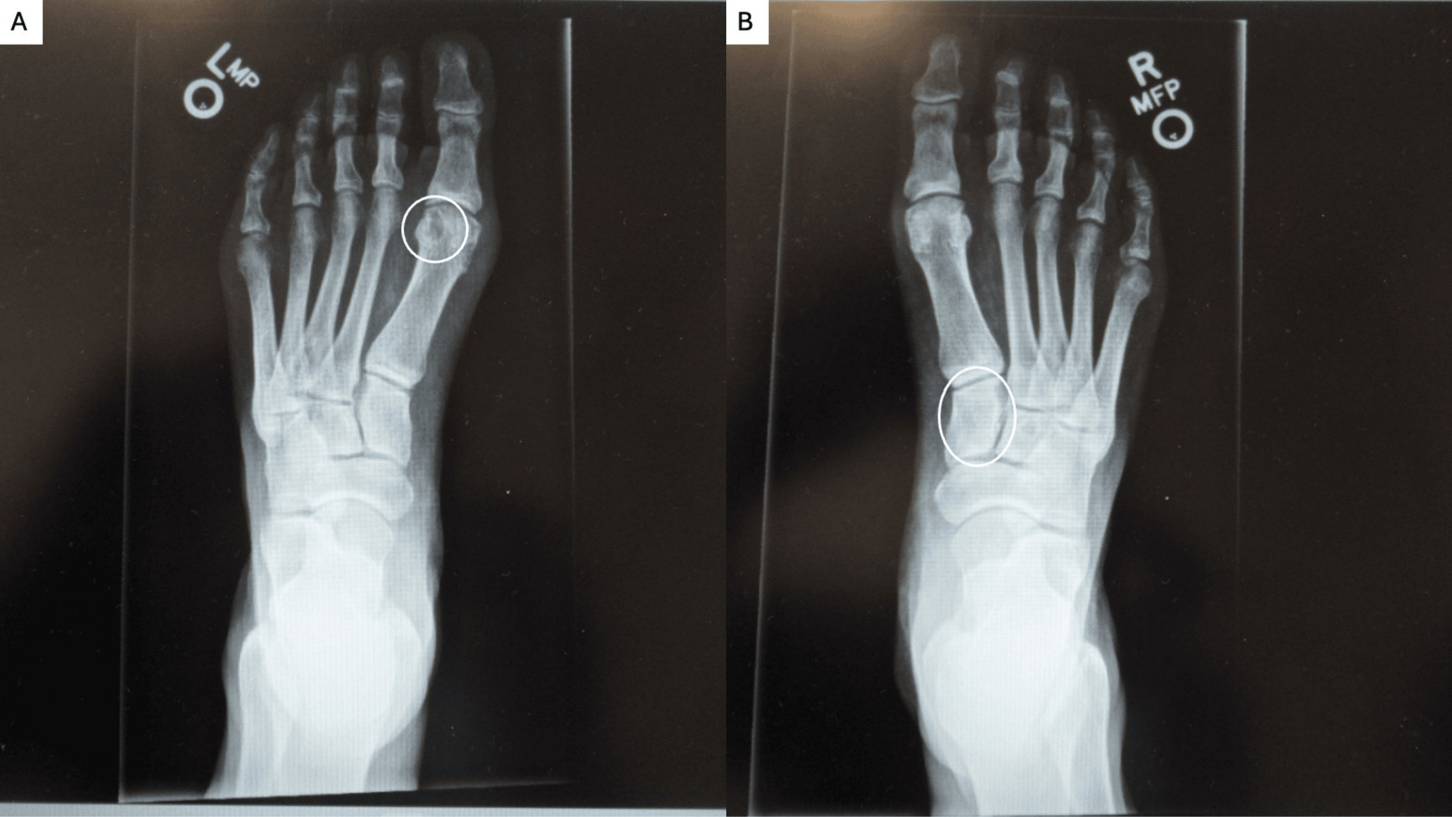
Radiograph of the patient's feet with gout. Roentgenograms showing the top view of the left (A) and right (B) foot. A well-defined erosion at the lateral aspect of the distal first metatarsal bone (within the hollow white oval) is noted on the left foot (A). A lucency, which was consistent with an erosion from a tophus, was present on the middle cuneiform (within the hollow white oval) of the right foot (B).

Bilateral foot films were also done at the appointment (Figure 5). The left foot had a well-defined erosion at the lateral aspect of the distal first metatarsal bone. The right foot had a lucency at the middle cuneiform; given the history of gout, this could represent an erosion from a tophus.

The correlation of the clinical presentation, the elevated uric acid level, and the changes observed on the radiographs of the feet established the diagnosis of gout. He was going to start a uricosuric medication, attempting to reach a serum uric acid of less than 5 mg/dL. He was also to take colchicine for prophylaxis to prevent a flare of gout flare while increasing the daily allopurinol dose.

He has begun on allopurinol 100 mg daily with colchicine 0.6 mg daily; he took the medications before going to sleep. After the first dose of the medications, he woke up with a sore throat. The same thing happened after the second and third doses; in addition, he had a fullness sensation in his throat. He stopped both medications and the symptoms resolved and did not recur.

His rheumatologist prescribed another xanthine oxidase inhibitor, febuxostat. He was reluctant to take this medication since febuxostat is linked to an increased risk of cardiac death, heart attack, stroke, and heart failure in some patients who take the medication; he had a history of atrial flutter and a stroke. Although not suggested, he would not have been able to take probenecid since this medication is a sulfonamide and patients with a history of sulfa drug allergy may have a hypersensitivity reaction to probenecid; he was allergic to trimethoprim/sulfamethoxazole.

Vitamin C may be helpful not only for treating acute and chronic gout but also for preventing recurrences of the disease. He began alternating 500 mg and 1000 mg of vitamin C each evening. He has not had another acute attack of gout since February 2024. If he does experience another acute episode, he will restart the five-day prednisone treatment.

## Discussion

Deposition of monosodium urate in and around the joints is frequently observed in individuals who have gout. Gout can progress through the following phases: asymptomatic hyperuricemia, monosodium urate crystal deposition, acute gout (including an arthritis attack), intercritical period (when the joints appear to have returned to normal after the resolution of the acute attack), and chronic gout (which is an advanced stage of the disease characterized by bony erosions, polyarticular arthritis, synovial hypertrophy, and tophi). The diagnosis of gout requires two of the following: at least two attacks (each of which resolves within two weeks of painful joint swelling, observation or a history of podagra (which is the involvement of the first metatarsal of the great toe) or the presence of a tophus, and prompt response (within 48 hours) after starting treatment with colchicine [1,4]. 

Hyperuricemia is usually present in patients with gout. Rheumatologic manifestations of gout include polyarticular arthritis, bony erosions, joint effusions, synovial hypertrophy, and tophi. In addition to the warm, tender, erythematous, swollen joint of an acute gout attack, cutaneous features of gout include nodular tophi, draining tophi, and chronic ulcers; other dermatologic presentations of gout, which have less commonly been observed, are disseminated cutaneous gout, gouty nodulosis, miliarial gout, panniculitis, and perforating gout [5,6].

There are two different treatment philosophies for managing gout. Rheumatologists (American College of Rheumatology, European Alliance of Associations for Rheumatology, and British Rheumatology Society) favor the “treat-to-target” with urate-lowering drug therapy until a specific serum urate level is reached. In contrast, the American College of Physicians advocates a strategy to “treat-to-avoid symptoms” and is not preoccupied with the individual’s specific serum urate level [7].

Advocates for treating hyperuricemia emphasize the risks associated with elevated levels of uric acid in the body are not only limited to gout. Indeed, the adverse effects of hyperuricemia also include cardiovascular disease, diabetes mellitus type II, hypertension, acute and chronic kidney injury, and urolithiasis. A serum uric acid above 12 mg/dL is an absolute indication for treatment [8].

Treatment independent of the serum uric acid level is urate-related urolithiasis and possibly gout. Asymptomatic hyperuricemia might warrant therapeutic intervention. The SCORE 2 scale is a valuable tool to assess cardiovascular risk that has been incorporated into the European Society of Cardiology 2021 guidelines [8].

The initial management of hyperuricemia includes lifestyle changes. These include eliminating dietary ingestion of foods that are high in purines such as meat, seafood, and offal (which includes not only the internal organs of animals such as kidney, heart, liver, lungs, pancreas, spleen, stomach, thymus, and thyroid but also the brains, feet, head, skin, tail, testicles, and tongue) [8]. It also includes cessation of alcohol (especially beer) and strong liquors; in addition, drinking non-diet soda that includes fructose should be eliminated [8]. However, diet alone is usually not successful in adequately lowering serum uric acid to an acceptable range [1].

Allopurinol, a xanthine oxidase inhibitor, is the first-line drug for gout therapy; usually, colchicine is initiated concurrently to prevent acute flares of gout [8,9]. Allopurinol has been associated with several hypersensitivity syndromes [8,10]. Severe cutaneous adverse reactions (SCARs) include Stevens-Johnson syndrome (SJS), toxic epidermal necrosis (TEN), drug reaction with eosinophilia and systemic symptoms (DRESS), and allopurinol hypersensitivity reaction (AHS) [8,10].

Evaluation for the presence of HLA-B5801 should be considered prior to initiating allopurinol therapy, particularly in individuals from East Asia [8]. Individuals who are HLA-B5801-positive are more susceptible to experience a hypersensitivity reaction to allopurinol [8]. The man in this report was HLA-B5801-negative. He became concerned that the throat symptoms he developed after starting the allopurinol and colchicine may have been an early prodrome to toxic epidermal necrolysis; this prompted him to stop both medications and not to restart them at a future date after the symptoms resolved.

In patients who cannot tolerate allopurinol or who do not respond to therapy, a noncompetitive nonpurine xanthine oxidase inhibitor (such as febuxostat) can be initiated for treatment; this drug does not cross-react with allopurinol [9,11,12]. However, the drug company that manufactures febuxostat introduced a new warning to the package insert that there is an increased risk of cardiac death, heart attack, stroke, and heart failure in some patients who take the medication [9]. The man in this report had a history of atrial flutter and cerebrovascular accident; therefore, he did not concur with his rheumatologist’s selection to initiate treatment with febuxostat.

Another effective drug for treating gout is pegloticase. Polyethylene glycol (PEG) is also referred to as macrogol; PEGylation refers to the process used to develop drugs by attaching strains of the polymer PEG. Pegloticase is a PEGylated uricase that is typically recommended to be administered once every two weeks as an intravenous infusion for gout [9].

Probenecid is a second-line therapy in the management of gout. It is not only a sulfonamide but also a uric acid transporter 1 (URAT1) agent that requires twice-daily dosing because of its short half-life. However, probenecid has a risk of drug-related urolithiasis and is contraindicated in patients with stage 4 chronic renal disease; in addition, as a single agent, it may not be effective in adequately lowering serum uric acid [12]. The man in this report had an allergy to trimethoprim/sulfamethoxazole manifested by urticaria; he was not going to take probenecid with his allergy history.

For acute attacks of gout, management typically involves anti-inflammatory medications. These can include NSAIDs, colchicine, or prednisone. For the man in this report, NSAIDs were contraindicated since he was on an oral anticoagulant; also, he developed throat symptoms after starting colchicine [5,11]. His acute gout flare promptly resolved during his five-day treatment with oral prednisone.

Dietary modification can lower serum uric acid levels and decrease the incidence of acute gout recurrent flares. The Western diet, which is high in animal protein, can exacerbate gouty inflammation. In contrast to the Western diet, diets that are higher not only in plant protein such as soybeans and soy products but also in low-fat dairy products are beneficial to lower hyperuricemia and prevent gout. Other diets that are beneficial in gout patients are characterized by calorie restriction and fasting, low purine content, and Mediterranean-style (primarily consisting of vegetables and fruits) [13].

Diets that are high in fat and sugar can induce hyperuricemia and inflammation. Metabolic disease can result from prolonged high sugar consumption. A diet consisting of high sugar content can also be accompanied by fat accumulation, hyperglycemia, and insulin resistance. Also, fructose-rich soft drinks (such as high-fructose corn syrup-sweetened non-diet sodas) can worsen gout; therefore, carbohydrate restriction (including limiting or eliminating fructose intake) is an effective dietary intervention for individuals with gout [13].

Vitamin supplements, fruits, and foods containing vitamins A, C, D, and E are good for the treatment of hyperuricemia and gout [13]. In addition, normal levels of minerals or electrolytes (such as calcium, copper, iron, potassium, selenium, and zinc) can lead to beneficial effects against gout [13]. There is a connection between fiber intake and an improvement of the gut microbiota to prevent intestinal flora disorder in gout patients [14]. Indeed, regulating gastrointestinal homeostasis results has beneficial effects (such as reducing uric acid production and inflammation) in gout patients created by the consumption of more fiber-rich whole grains, vegetables, and fruits [13].

A concern for gout patients is the potential drug-drug interactions and medication-related adverse events; therefore, some individuals with gout use alternative and complementary medicine in the management of their condition [15]. In a study of 276 patients with gout, 66 (24%) of the patients reported using one or more alternative and complementary medicines; the most used modalities were dietary supplements (38 users), vitamins (16 users), and herbal medicines (15 users). Other less often used modalities included heat treatment, massage therapy, spiritual healing, topical ointments, aromatherapy, naturopathy, homeopathy, and ayurvedic medicine [15].

Acupuncture has been used to treat gouty arthritis in East Asian countries. The nonpharmacologic technique is inexpensive to perform and has a favorable side effect profile. In addition to the traditional manual acupuncture approach, other forms of the technique include electro-acupuncture, acupotomy, and fire-needling acupuncture [16].

Herbal medicines for external use have been incorporated into Korean medicine (including herbal bath therapy and external patches) and traditional Chinese medicine for the treatment of gout. Reduction of uric acid levels, pain intensity, and inflammation have been observed when patients with gout have been treated with herbal medicines for external use alone or in combination with Western drugs. Importantly, the herbal medicines for external use achieved greater efficacy than the Western drugs; in addition, the topical medicines did result in a serious risk for the patient. Therefore, for the treatment of pain and symptoms of acute gouty arthritis, herbal medicines for external use can be a safe and effective therapeutic alternative [17].

Cherries may have a role in the prevention of recurrent flares of gout and the management of acute and chronic disease; they contain vitamins A, C, and E. They also contain anthocyanins, which are water-soluble pigments that possess both antioxidant and anti-inflammatory properties. In addition, cherries have hypouricemic effects and the ability to downregulate nuclear factor-kappa B-mediated osteoclastogenesis [18].

The acute and chronic inflammation associated with recurrent gout flares may be reduced by cherries. In addition, the chronic destructive arthropathy of gout may be reduced by cherries. Indeed, cherries or cherry products (such as cherry concentrates, cherry extract, cherry juice, and cherry powder) have been used by approximately 25% of gout patients [18].

In a survey study of 633 gout patients, cherry intake over a two-day period resulted in a 35% lower risk of gout attack or gout flare. In another study, 20 gout patients received a tablespoon (15 mm) of cherry juice concentrate (which was the equivalent of 45-60 cherries) twice daily for four months; the number of flares in nine of the patients was reduced from 5 to 1.5, and the remaining 11 patients were gout flare-free. In a third study of 24 patients who took cherry concentrate for four months or longer as chronic gout flare prophylaxis, the number of gout flares decreased from nearly seven to two [18]. Cherries contain salicylates, which can potentially cause blood thinning; therefore, the man in this report did not elect to eat an increased number of cherries to try to prevent his gout from recurring.

Vitamin C, L-ascorbic acid, has repeatedly been demonstrated to be uricosuric. It acts on multiple aspects of the nephron, including the proximal tubules and the glomeruli. In addition, vitamin C reduced the incidence of gout by decreasing the monosodium urate deposition-associated nuclear factor-kappa B/nucleotide-binding domain, leucine-rich repeat, and pyrin domain-containing protein 3-related inflammatory response. Vitamin C supplementation can reduce hyperuricemia or prevent incident and recurrent gout [13,19,20].

A large prospective study reported in 2009 consisted of 20 years of follow-up and included 1317 patients with gout; the study demonstrated that higher vitamin C intake was independently associated with a lower risk of gout. The daily dose ranged from less than 250 mg to more than 1500 mg. The investigators observed that a total vitamin C intake of 500 mg daily or more was associated with a reduced risk of gout and concluded that supplemental vitamin C intake may be beneficial in the prevention of gout [19].

In 2021, another meta-analysis of randomized controlled trials evaluating the association between oral vitamin C supplementation and serum uric acid was performed. In comparison to the paper from 2011, the current investigators were more definitive in their interpretation of the data. They included 16 eligible trials, containing 1013 participants, and determined that the pooled findings showed that vitamin C supplementation had a significant effect on lowering serum uric acid [20].

The recommended dietary allowance of vitamin C is 75 mg per day in women and 90 mg per day in men. All 16 studies each had an intervention dose (200-2000 mg per day) above the recommended daily dose of vitamin C. However, when the investigators analyzed the data, they found that there was no difference in benefit whether the dose was less than 500 mg or greater than 500 mg; they could not confirm an advantage or disadvantage of using a higher daily dose [20]. The reported patient decided to alternate 500 mg and 1000 mg of vitamin C each evening.

The investigators found that the effects of the supplemental vitamin C intervention alone were better than that of vitamin C combined with other drugs. The researchers attributed this to a possible interaction of vitamin C with the uricosuric drugs [20]. The reported man was pleased with this observation since he was not taking any specific gout-related medication.

The researchers noted that the baseline level of the serum uric acid had no effect on the intervention of vitamin C. Also, they found that vitamin C supplementation was more effective in individuals younger than 65 years of age [20]. Since the patient was already 65 years old, he was disappointed by this observation.

## Conclusions

Gout, a disorder of uric acid metabolism, may present with pathognomonic clinical features such as podagra. A 65-year-old man with gout-related podagra could not tolerate allopurinol and colchicine; in addition, he could not be treated with febuxostat or probenecid. Vitamin C has been demonstrated not only to lower serum uric acid levels but also to reduce the risk of incident and recurrent gout. He began vitamin C (at a daily dose alternating between 500 mg and 1000 mg); he has not had a recurrent acute gout attack in nearly one year. In conclusion, several systemic medications are recommended for the treatment and prevention of gout; however, in individuals for whom these drugs are ineffective or contraindicated, alternative and complementary medicines may be considered.

## References

[1] Mikuls TR (2022). Gout. N Engl J Med.

[2] (2025). NatureLife: Gout and the famous people who suffer from it. https://www.naturelife.co.za/2018/05/26/gout-and-the-famous-people-who-suffer-from-it/.

[3] Rothschild BM, Tanke D, Carpenter K (1997). Tyrannosaurs suffered from gout. Nature.

[4] Low QJ, Lim TH, Hon SA, Low QJ, Wei MW, Cheo SW, Ramlan AH (2022). Management of gout in the primary care setting. Malays Fam Physician.

[5] Cohen PR (2024). What caused this forearm nodule?. The Dermatologist.

[6] Cohen PR, Schmidt WA, Rapini RP (1991). Chronic tophaceous gout with severely deforming arthritis: a case report with emphasis on histopathologic considerations. Cutis.

[7] Stamp LK, Dalbeth N (2024). Moving urate-lowering therapy in gout beyond guideline recommendations. Semin Arthritis Rheum.

[8] Domański I, Kozieł A, Kuderska N, Wójcik P, Dudzik Ł, Dudzik T (2024). Hyperuricemia - consequences of not initiating therapy. Benefits and drawbacks of treatment. Reumatologia.

[9] Quintana MJ, Shum AZ, Folse MS (2023). Gout treatment and clinical considerations: the role of pegloticase, colchicine, and febuxostat. Cureus.

[10] Dillman KM, Hawkins AM, Ragland AR (2024). Allopurinol: clinical considerations in the development and treatment of Stevens-Johnson syndrome, toxic epidermal necrolysis, and other associated drug reactions. Cureus.

[11] Zeng X, Liu Y, Fan Y, Wu D, Meng Y, Qin M (2023). Agents for the treatment of gout: current advances and future perspectives. J Med Chem.

[12] Terkeltaub R (2023). Emerging urate-lowering drugs and pharmacologic treatment strategies for gout: a narrative review. Drugs.

[13] Zhang Y, Chen S, Yuan M, Xu Y, Xu H (2022). Gout and diet: a comprehensive review of mechanisms and management. Nutrients.

[14] Wen YF, Culhane-Pera KA, Pergament SL (2023). Hmong microbiome and gout, obesity, vitamin C (HMANGO-C): a phase II clinical study protocol. PLoS One.

[15] Chan E, House ME, Petrie KJ, Horne A, Taylor WJ, Dalbeth N (2014). Complementary and alternative medicine use in patients with gout: a longitudinal observational study. J Clin Rheumatol.

[16] Lee G, Cho FY, Goo B, Park YC (2020). Acupuncture for gouty arthritis: a PRISMA-compliant protocol for a systematic review and meta-analysis of randomized controlled trials. Medicine (Baltimore).

[17] Choi SH, Song HS, Hwang J (2023). Herbal medicine for external use in acute gouty arthritis: a PRISMA-compliant systematic review and meta-analysis. Medicine (Baltimore).

[18] Collins MW, Saag KG, Singh JA (2019). Is there a role for cherries in the management of gout?. Ther Adv Musculoskelet Dis.

[19] Choi HK, Gao X, Curhan G (2009). Vitamin C intake and the risk of gout in men: a prospective study. Arch Intern Med.

[20] Liu XX, Wang XX, Cui LL (2021). Association between oral vitamin C supplementation and serum uric acid: a meta-analysis of randomized controlled trials. Complement Ther Med.

